# Assessing Statistical Methods for Estimating the Cerebrospinal Fluid Early Fungicidal Activity as an Endpoint for Cryptococcal Meningitis Clinical Trials: A Systematic Review and Simulation Study

**DOI:** 10.1093/ofid/ofag159

**Published:** 2026-03-22

**Authors:** Monica Fuszard, David R Boulware, Biyue Dai

**Affiliations:** Division of Biostatistics and Health Data Science, School of Public Health, University of Minnesota, Minneapolis, Minnesota, USA; Division of Infectious Diseases and International Medicine, Department of Medicine, University of Minnesota, Minneapolis, Minnesota, USA; Division of Biostatistics and Health Data Science, School of Public Health, University of Minnesota, Minneapolis, Minnesota, USA

**Keywords:** cryptococcal meningitis, early fungicidal activity, linear mixed models, simulation, systematic review

## Abstract

**Background:**

Cryptococcal meningitis is a life-threatening infection of the central nervous system. Early fungicidal activity (EFA), which measures how quickly *Cryptococcus* yeasts are cleared from cerebrospinal fluid (CSF) over time, is a key endpoint in phase 2 antifungal clinical trials. EFA is commonly estimated via a 2-step approach, where simple linear regressions are first fit to each individual's longitudinal measurements, and the slopes are then aggregated for statistical inference. Alternatively, a linear mixed model can estimate individual and group-level effects in one model. A systematic comparison of these methods is needed to understand and clarify their differences in analyzing EFA data in clinical trials.

**Methods:**

We conducted a systematic literature review to summarize the usage and reporting of both methods in cryptococcal meningitis clinical trials. We then evaluated their performance and operating characteristics under various simulation scenarios and applied both approaches to data from a phase 2 trial.

**Results:**

The 2-step approach and mixed model approach yielded discrepant CSF EFA estimates across literature review, simulation experiments, and real data analysis. The 2-step approach using linear regression produced steeper estimates and larger estimated treatment effects. The linear mixed model yielded smaller standard errors but estimates were biased toward the null, especially when early culture sterility was common.

**Conclusions:**

The 2-step approach is preferred for estimating EFA in cryptococcal meningitis studies, especially when early culture sterility occurs. EFA estimates should not be interpreted or compared across studies without accounting for the underlying statistical models and data preprocessing.

Cryptococcal meningitis is a life-threatening disseminated infection of the spinal fluid, brain, and throughout the body that primarily affects people with weakened immune systems, such as those with advanced human immunodeficiency virus (HIV). Cryptococcosis accounts for 19% (13%–24%) of AIDS-related mortality globally [1], with most cases and deaths occurring in resource-limited settings [2].

Per the current World Health Organization guidelines [3], the clinical management of cryptococcal meningitis includes 3 phases: a 1- to 2-week induction phase, an 8-week consolidation phase, and then secondary prophylaxis [4]. In phase 2 cryptococcal meningitis clinical trials, the standard primary endpoint has been the rate of fungal clearance in cerebrospinal fluid (CSF) during the induction phase of treatment, often referred to as early fungicidal activity (EFA) [5]. In phase 3 confirmatory trials, all-cause mortality often serves as the primary endpoint to obtain a comprehensive assessment of safety and efficacy. EFA, which only involves data collection during the 2-week induction phase, is a key factor in assessing whether the new antifungal regimens should be moved forward to a phase 3 confirmatory trial, which requires a much larger sample size, time, and resources to conduct. EFA has also been evaluated as surrogate endpoint for overall mortality [6, 7]

To calculate EFA, CSF samples first need to be obtained through serial lumbar punctures over time. The CSF fungal burden will then be quantified using 10-fold serial dilutions of CSF to measure the number of colony-forming units (CFU) of *Cryptococcus* per milliliter of CSF [8]. A log_10_ transformation is typically applied to the CSF quantitative culture as a data preprocessing step both to normalize the data as well as due to logarithmic antifungal clearance. Since its initial proposal, EFA has been commonly calculated using a 2-step approach. The log_10_-transformed quantitative culture measurements from each individual are fit with a simple linear regression in the first step, and the estimates of the individual slopes are then aggregated in the second step for further statistical inference. Recent cryptococcal trials have also reported EFA estimates via linear mixed models (LMMs) [9–13], which handle the individual-level estimation and group-level estimation in a single model.

The 2-step approach and the one-step mixed model approach both appropriately handle the within-participant correlations for repeated measurements under certain statistical assumptions and have been studied in other contexts that involve data with hierarchical structures, such as cluster randomized trials and individual patient data meta-analysis [14, 15]. However, in the context of modeling fungal clearance rate for cryptococcal meningitis studies, there remain open questions as to the empirical performance of these 2 methods on the CSF fungal clearance data, how to interpret the discrepancies in EFA estimates from the 2 models, and whether those discrepancies may lead to differences in clinical interpretations and decisions.

We aimed to answer the above questions with a mixed-methods approach. We first conducted a systematic literature review on statistical methods and EFA estimates reported in published cryptococcal meningitis studies. We then conducted simulation experiments in the context of both single-arm and randomized studies. Specifically, we empirically assessed operational characteristics including bias, power, type I error, coverage probabilities, and lengths of confidence intervals (CIs) under various realistic scenarios where some model assumptions can be violated. Finally, we analyzed CSF culture data from a phase 2 cryptococcal clinical trial to assess the models’ performance on real data.

## METHODS

### Systematic Review

The search for relevant literature was conducted using PubMed, Science Direct, and ClinicalTrials.gov in August 2025. Search terms included “HIV,” “cryptococcal meningitis,” and “early fungicidal activity.” A reference list of 15 keystone articles was used in the generation of relevant sources. Three categories of articles were considered: (1) major publications for clinical trials evaluating cryptococcal meningitis treatment, (2) secondary data analyses using data from prior cryptococcal clinical trials, and (3) methods-related publications for the analysis of EFA data. Articles were included as findings if they used simple linear regression, an LMM, or another specified statistical model in the estimation of EFA.

### Statistical Approaches of Interest

#### Two-Step Approach

In the 2-step approach for estimating EFA, a simple linear regression will first be fitted for each participant *i*, with the log_10_-transformed quantitative CSF culture in CFU/mL as the independent variable $Y_{i\;}$, and time as the dependent variable *X*:


$$Y_{i}=\beta_{i0}+\beta_{i1}X+\varepsilon$$

The intercept $\beta_{i0}$ represents the *i*th participant's fungal burden at baseline. The slope $\beta_{i1}$ represents the rate of decline in fungal burden over time, and a steeper slope indicates a faster clearance. The error term ε assumes to be identically and independently normally distributed. In the second step, the individual EFA estimates ${\hat{\beta }}_{i1}$ can be aggregated to draw group-level inference. The sample mean and sample mean difference with their 95% CIs can be used to estimate treatment effects. A 2-sample *t*-test over the individual-level slopes can be used to make statistical comparisons between treatment groups.

#### Linear Mixed Models

LMMs simultaneously estimate individual-level and group-level effects, accounting for correlation among repeated measurements within participants [16]. The LMM can be expressed as:


Y=βX+γZ+ϵ,

where *Y* constitutes the quantitative cultures for all participants and *X* and *Z* represent the design matrices for fixed and random effects, and β and γ denote their corresponding coefficients. In the context of estimating EFA, like the simple linear regression model, intercepts capture participants’ baseline fungal burden, while slopes, which represent the fungal clearance rate, provide estimates for EFA. Participant-level EFA can be derived from a combination of fixed and random slopes, while group-level EFA can be obtained from the marginal mean estimate. When there are multiple treatment arms in the study, treatment arm should be fitted as a fixed effect, with an interaction term with time, to enable the by-arm estimation of fungal clearance rate. For this manuscript, LMMs were fitted using R package lme4 [17], and marginal mean estimates and corresponding standard errors were extracted using R package emmeans [18].

### Simulation Studies

We conducted simulation studies to evaluate the performance of the 2-step and LMM approaches under various conditions. Artificial datasets were generated to mimic the characteristics and structures of real EFA data. The following metrics and operational characteristics were used to assess the performance: Statistical bias quantifies whether a method consistently overestimates or underestimates the reality, which is represented by the true value of the EFA in the data-generating model. Length of the 95% CI quantifies how precise and variable the estimates are. Coverage probability of 95% CI quantifies the probability that the true value is contained within the 95% CI. Low coverage probability typically indicates that the method misses the target and is likely biased. Typically, there is a trade-off between the length and the coverage probability of the CI. Type I error rate is the probability of incorrectly concluding that a statistical difference exists when it does not. If a statistical analysis is conducted with an alpha of .05, then the desirable type I error rate is at 0.05. Statistical power is the probability that a true statistical difference can be detected by the method of interest. Effect size is the magnitude of the treatment difference between the 2 groups to be detected, and smaller effect sizes are harder to detect. Given the same sample size, a more powerful statistical method can detect a smaller effect size.

#### Data-Generating Process

For each simulated data set, log_10_-transformed CSF quantitative culture trajectories were generated for a prespecified number of participants. For each individual trajectory, the slope and intercept pairs were first generated using a multivariate probability distribution, which allows for the generation of correlated pairs. Both the multivariate normal distribution [19] and the multivariate lognormal distribution were used to better mimic the skewed nature of the real-world data [20]. Once slope and intercept pairs were obtained, they were then used to generate the fungal burden at baseline and 4 subsequent time points for each participant. Random noises were added to the fungal burden value at each time point with the standard normal distribution. Each parameter in the data generation process was fine-tuned to mimic real EFA data observed in cryptococcal clinical trials, in terms of mean, correlation, variability, and skewness. Therefore, additional preprocessing was performed on the simulated data, such as replacing instances of negative values with 0, removing all cases that were sterile at baseline, and removing repeat sterile observations. Once the simulated data are generated and processed, models described above were fitted to extract EFA estimates.

#### Simulation Settings

Simulations were conducted to assess the performance of both models in 2 common trial settings: a single-arm trial with a clinically meaningful historical threshold and a 2-arm randomized trial with an experimental arm and a control arm. In both settings, simulations were used to assess type I error and power, using a multivariate normal distribution as well as a multivariate lognormal distribution. Eight simulations were conducted to assess the power of both models with varying sample size and effect size. This process was then repeated for the 2-sample test comparing concurrent treatment groups.

To investigate the impact of proportions of early sterility, an additional simulation experiment was designed. In the literature, once a participant reaches sterility, the next repeat sterile values are typically removed during data preprocessing. Therefore, participants who reach sterility within the 2-week window tend to have fewer measurements. In this experiment, we generated data using a fixed EFA value with varying baseline fungal burden. In this way, we artificially created datasets with varied proportion of participants with early sterility, while controlling for the EFA value. We then fit both models for the estimation of EFA in each simulated dataset to assess bias of the EFA estimators.

R version 4.2.0 software was used for all statistical analyses.

### Analysis of Real Data

Data for this analysis were obtained from the Encochleated Oral Amphotericin for Cryptococcal Meningitis Trial (ENACT) [13], which compared oral amphotericin, a novel formulation of amphotericin, with the traditional amphotericin B deoxycholate given intravenously for HIV-associated cryptococcosis. All participants received lumbar punctures at diagnosis, day 3, days 5–7, days 10–14, and additionally as required for intracranial pressure management and CSF sterilization assessment.

## RESULTS

### Literature Review

**Figure 1:**
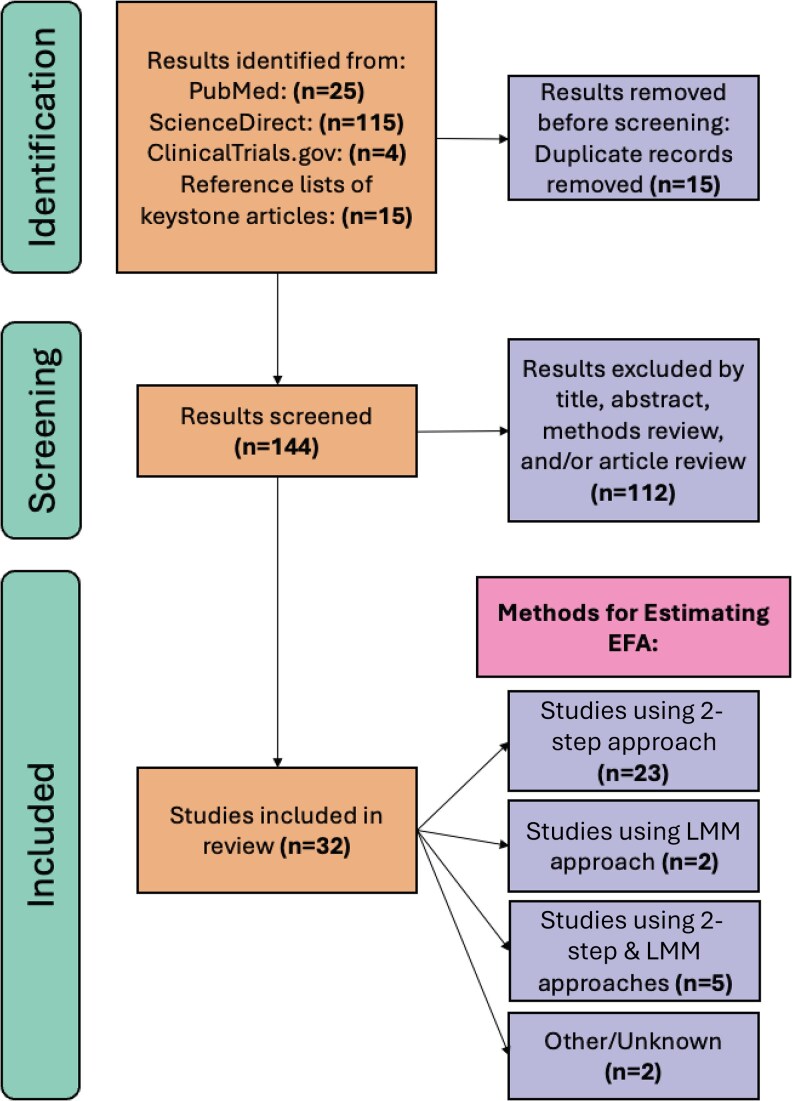
Flow diagram of studies included in the systematic review of statistical methods used for fungal clearance in analyzing cryptococcal meningitis. Abbreviations: EFA, early fungicidal activity; LMM, linear mixed model.

The systematic review of literature produced 32 relevant sources that span 2 decades of cryptococcal research (Table 1). Most sources used the 2-step approach to estimate EFA (Figure 1). The LMM as a tool for estimating EFA was not seen in the literature until the 2013 analysis of a randomized controlled trial of combination antifungal therapy by Day et al [33]. Since then, there have been 7 studies that implemented an LMM in the estimation of EFA, 5 of which used both the 2-step approach and an LMM to produce estimates of EFA. In each of these studies, the LMM estimates for EFA were consistently more attenuated compared to the estimates produced by the 2-step approach (Supplementary Table 1). All 5 studies were transparent in terms of reporting the EFA results and which models were used, and they were all designed as randomized trials with additional clinical outcomes such as all-cause mortality. The key clinical signals and conclusions within each study remain consistent regardless of the method used for EFA estimation. Removing repeat observations of sterility after a participant reaches sterility is a common data preprocessing step [10, 11].

**Table 1.** Listing of Studies Included in the Systematic Review

Authors, Year	Treatment Group(s)	No.	EFA Estimate^a^ (2-Step Approach of Linear Regression)	EFA Estimate^a^ (LMM/Other Approach)	Study Type	Model(s) Used
Brouwer et al, 2004 [5]	Amphotericin B	14	0.31 (0.18)	…	Clinical trial	2-step approach
	Ampho + 5FC	12	0.54 (0.19)	…		
	Ampho + fluconazole	11	0.39 (0.15)	…		
	Ampho + 5FC + fluconazole	15	0.38 (0.13)	…		
Brouwer et al, 2007 [21]	Ampho + 5FC	27	0.46 (0.18)	…	Clinical trial	2-step approach
	Ampho, no 5FC	25	0.34 (0.17)	…		
	Ampho + oral 5FC	13	0.48 (0.22)	…		
	Ampho + IV 5FC	14	0.43 (0.13)	…		
Bicanic et al, 2007 [22]	Ampho 1 mg/kg	49	0.48 (0.28)	…	Prospective observational study	2-step approach
	Fluconazole 400 mg	5	0.02 (0.05)	…		
Bicanic et al, 2008 [23]	Ampho 1 mg/kg + 5FC	34	0.56 (0.24)	…	Clinical trial	2-step approach
	Ampho 0.7 mg/kg + 5FC	30	0.45 (0.16)	…		
Longley et al, 2008 [24]	Fluconazole 1200 mg	30	0.18 (0.11)	…	Clinical trial	2-step approach
	Fluconazole 800 mg	30	0.07 (0.17)	…		
Bicanic et al, 2009 [25]	Non-IRIS	54	0.47 (0.17)	…	Prospective observational study	2-step approach
	IRIS	11	0.50 (0.25)	…		
Bicanic et al, 2009 [26]	Combined cohorts	262	0.37 (0.27)	…	Secondary analysis	2-step approach
Nussbaum et al, 2010 [27]	Fluconazole 1200 mg	20	0.11 (0.09)	…	Clinical trial	2-step approach
	Fluconazole 1200 mg + 5FC	21	0.28 (0.17)	…		
Loyse et al, 2012 [28]	Ampho + 5FC	21	0.41 (0.22)	…	Clinical trial	2-step approach
	Ampho + fluconazole 800 mg	22	0.38 (0.18)	…		
	Ampho + fluconazole 1200 mg	23	0.41 (0.35)	…		
	Ampho + voriconazole	13	0.44 (0.20)	…		
Muzoora et al, 2012 [29]	Ampho (5 d) + fluconazole 1200 mg	30	0.31 (0.11)	…	Clinical trial	2-step approach
Jackson et al, 2012 [30]	Ampho (7 d) + fluconazole 1200 mg	19	0.38 (0.19)	…	Clinical trial	2-step approach
	Ampho (7 d) + fluconazole 1200 mg + 5FC	18	0.50 (0.15)	…		
Jarvis et al, 2012 [31]	Ampho + 5FC	30	0.49 (0.17)	…	Clinical trial	2-step approach
	Ampho + 5FC + IFN-γ	60	0.64 (0.27)	…		
Bisson et al, 2013 [32]	Intervention: Ampho + ART within 7 d	13	0.32 (0.20)	…	Clinical trial	2-step approach
	Control: Ampho + ART after 28 d	14	0.52 (0.48)	…		
Day et al, 2013 [33]	Ampho	99	…	0.31 (.29–.34)	Clinical trial	LMM approach
	Ampho + 5FC	100	…	0.42 (.40–.44)		
	Ampho + fluconazole 800 mg	99	…	0.32 (.34–.29)		
Jarvis et al, 2013 [34]	IFN-γ/TNF-α predominant response	20	0.64	…	Secondary analysis	2-step approach
	No IFN-γ/TNF-α–predominant response	24	0.52	…		
Boulware et al, 2014 [46]	Ampho + fluconazole (800 mg/d)	189	0.36 (.32–.40)	0.31 (.28–.33)	Clinical trial	2-step & LMM approaches
Jarvis et al, 2015 [35]	…	…	…	…	Secondary analysis	2-step approach
Dyal et al, 2016 [8]	Quantitative culture technique: St George's method	378	0.45 (.33–.56)	…	Secondary analysis	2-step approach
	Quantitative culture technique: ACTG	229	0.40 (.29–.51)	…		
Beardsley et al, 2016 [36]	Dexamethasone	224	…	0.21 (.19–.24)	Clinical trial	LMM approach
	Placebo	226	…	0.31 (.28–.34)		
Rhein et al, 2016 [37]	Ampho + fluconazole (800 mg/d) + sertraline	128	0.43 (.37–.50)	0.37 (.33–.41)	Clinical trial	2-step & LMM approaches
Beardsley et al, 2019 [38]	…	…	…	…	Secondary analysis	Unknown
Molloy et al, 2018 [9]	Treatment difference: oral regimen vs 2-wk Ampho	…	0.16 (.12–.21)	0.10 (.07–.13)	Clinical trial	2-step & LMM approaches
	Treatment difference: 1-wk Ampho vs 2-wk Ampho	…	0.02 (−.02 to .07)	0.01 (−.01 to .04)		
Villanueva-Lozano et al, 2018 [39]	Ampho + fluconazole 800 mg + sertraline	7	0.29 (0.083)	…	Clinical trial	2-step approach
	Ampho + fluconazole 800 mg + placebo	5	0.25 (0.083)	…		
Jarvis et al, 2019 [40]	Control	17	0.41 (.36–.47)	…	Clinical trial	2-step approach
	Single-dose L-AmB	16	0.52 (.33–.71)	…		
	2-dose L-AmB	18	0.47 (.32–.60)	…		
	3-dose L-AmB	18	0.54 (.33–.76)	…		
Rhein et al, 2019 [12]	Ampho + fluconazole 800 mg + sertraline	229	0.43 (.37–.50)	0.33 (.30–.36)	Clinical trial	2-step & LMM approaches
	Ampho + fluconazole 800 mg + placebo	231	0.47 (.40–.54)	0.33 (.30–.35)		
Katende et al, 2019 [41]	Fluconazole 1200 mg + sertraline 400 mg	10	0.26 (.11–.42)	…	Clinical trial	2-step approach
	Fluconazole 1200 mg + sertraline + Ampho 5 d	13	0.47 (.34–.60)	…		
Ngan et al, 2021 [11]	Ampho + fluconazole	26	…	0.48 (.37–.61)	Clinical trial	Other approach (joint survival and LMM)
	Ampho + fluconazole + tamoxifen	24	…	0.49 (.37–.62)		
Jarvis et al, 2022 [10]	IV Ampho deoxycholate + 5FC	381	0.44 (0.21)	0.42 (0.13)	Clinical trial	2-step & LMM approaches
	L-AmB + 5FC + fluconazole	363	0.41 (0.19)	0.40 (0.13)		
Boulware et al, 2023 [13]	Oral Ampho with 2 IV loading doses	37	0.42 (.29–.55)	0.33 (.27–.39)	Clinical trial	2-step & LMM approaches
	All-oral Ampho	33	0.40 (.17–.64)	0.25 (.19–.31)		
	IV Ampho, Controls	34	0.46 (.36–.55)	0.37 (.31–.44)		
Stott et al, 2024 [42]	Control	31	Median: 0.30 (IQR, 0.14–0.40)	…	Secondary analysis	2-step approach
	Single-dose L-AmB	33	Median: 0.32 (IQR, 0.20–0.40)	…		
Gakuru et al, 2025 [43]	AMBITION-cm Trial Cohort	171	0.39 (.35–.45)	…	Secondary analysis	2-step approach
	Observational cohort	179	0.42 (.31–.53)	…		
Kimuda et al, 2025 [44]	L-AmB	46	0.50 (.36–.63)	…	Secondary analysis	2-step approach
	Ampho deoxycholate	155	0.40 (.36–.45)	…		

^a^Plus–minus values are mean (standard deviation). Otherwise, values are mean (95% confidence interval) unless otherwise indicated.

Abbreviations: 5FC, Flucytosine; ACTG, AIDS Clinical Trials Group; Ampho, Amphotericin B; ART, antiretroviral; EFA, early fungicidal activity in log_10_ colony-forming units/mL/day; IFN-γ, interferon gamma; IQR, interquartile range; IRIS, immune reconstitution inflammatory syndrome; IV, intravenous; L-AmB, liposomal Amphotericin B; LMM, linear mixed model; TNF-α, tumor necrosis factor alpha.

### Simulation Studies

#### Single-Arm Trial With Historical Benchmark Scenario

**Figure 2.**
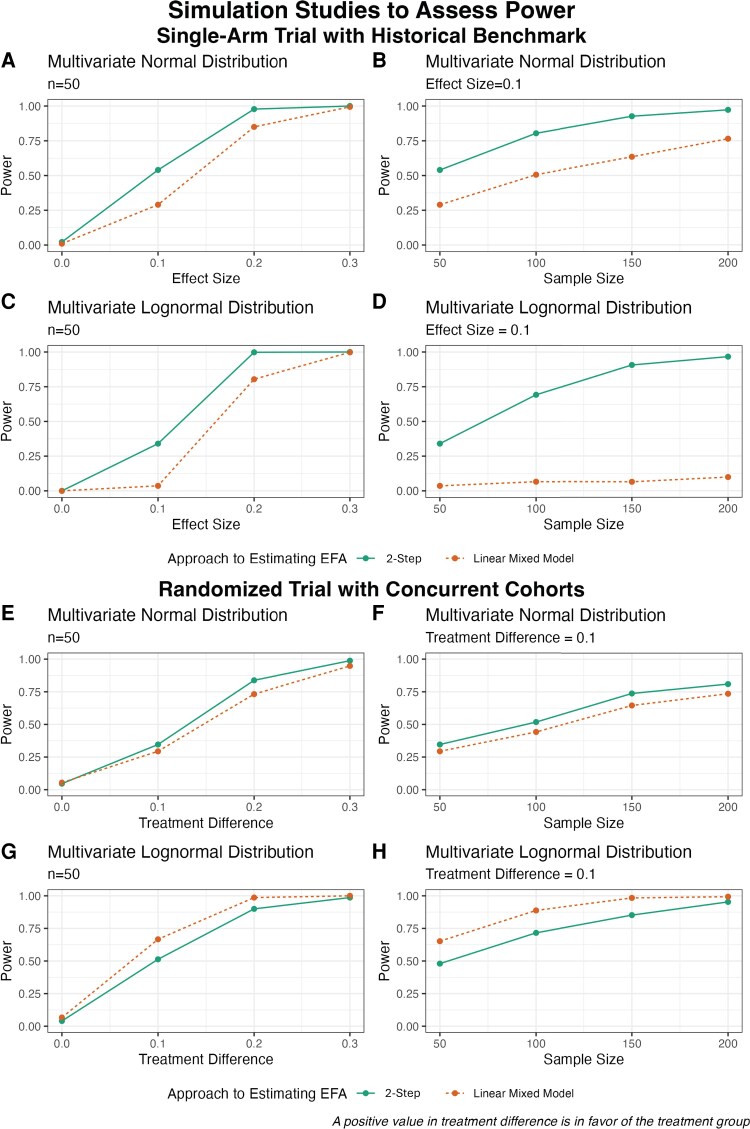
Simulated power curves for the 2-step approach and linear mixed model (LMM) approach, varying treatment effects, sample sizes, and underlying distribution. Panels *A–D* are based on historical benchmark scenario, with effect size increases from 0.0 to 0.3 in *A* and *C*, and sample size increases from 50 to 200 in *B* and *D*. The 2-step approach consistently demonstrated higher power than the LMM approach. Panels *E–H* are based on concurrent cohorts scenarios, with treatment difference increases from 0.0 to 0.3 in *E* and *G*, and sample size increases from 50 to 200 in *F* and *H*. The 2-step method and LMM perform similarly across *E–H*, with the 2-step method generally performing better under normal data (*E* and *F*) and LMM performing better under lognormal data (*G* and *H*). Abbreviation: EFA, early fungicidal activity.

Across all simulations of single-arm cryptococcal trials comparing EFA against a historical benchmark, the LMM produces an attenuated estimate of EFA compared to the 2-step approach (Table 2). Case 1 illustrates that when simulated log_10_ quantitative culture values are generated from the multivariate normal distribution and both the true simulated EFA value and the historical benchmark are null, both LMM and the 2-step approach provide estimates of EFA that are quite close to the true value, and LMM has a slightly greater coverage probability and narrower CI than the 2-step approach. However, when the true simulated EFA value is not null, the LMM is overly conservative in its estimation of EFA and produces more biased estimates compared to the 2-step approach. This is also the case when log_10_ quantitative culture values are simulated using the multivariate normal distribution (Case 2) or the multivariate lognormal distribution (Case 3) (Table 2). Although LMM produces narrower CIs than the 2-step approach, this does not fully compensate for the biased estimates, which resulted in lower coverage probability than the 2-step approach. Power simulations under varied conditions also show that the 2-step approach consistently outperforms LMM when comparing estimated EFA against a historical benchmark (Figure 2*A*–*D*). Across each scenario, we see a consistent loss of power in the LMM compared to the 2-step approach.

**Table 2.** Simulation Results for Historical Benchmark and Concurrent Cohorts Scenarios

Simulation	Mean	Median	Type I Error	Coverage	CI Width
Estimated EFA^a^—historical benchmark simulation results					
Case 1: Multivariate normal distribution, historical benchmark = 0, true mean parameter = 0					
True value	−0.003	−0.001	…	…	…
2-step	−0.015	−0.017	0.056	0.926	0.167
Linear mixed model	−0.014	−0.015	0.052	0.930	0.161
Case 2: Multivariate normal distribution, historical benchmark = −0.2, true mean parameter = −0.2					
True value	−0.203	−0.201	…	…	…
2-step	−0.195	−0.198	0.022	0.942	0.158
Linear mixed model	−0.181	−0.183	0.010	0.922	0.148
Case 3: Multivariate lognormal distribution, historical benchmark = −0.2, true mean parameter = −0.2					
True value	−0.195	−0.111	…	…	…
2-step	−0.173	−0.171	0	0.758	0.125
Linear mixed model	−0.138	−0.138	0	0.140	0.078
Estimated treatment difference^b^—concurrent cohorts simulation results					
Case 1: Multivariate normal distribution, true mean parameter = 0 in both cohorts					
2-step	0.003	0.005	0.052	0.948	0.234
Linear mixed model	0.003	0.005	0.056	0.944	0.228
Case 2: Multivariate normal distribution, true mean parameter = −0.2 in both cohorts					
2-step	0.003	0.004	0.046	0.954	0.224
Linear mixed model	0.001	0.002	0.054	0.946	0.210
Case 3: Multivariate lognormal distribution, true mean parameter = −0.2 in both cohorts					
2-step	0.002	0.002	0.052	0.948	0.182
Linear mixed model	0.002	0.001	0.062	0.938	0.110

For the 2-step approach, the 95% CIs are estimated directly from the individual simple linear regression slopes. For the linear mixed model approach, marginal slopes and corresponding standard errors of the linear mixed model are obtained using lsmeans function in R and are used to calculate a 95% CI.

Abbreviations: CI, confidence interval (95%); EFA, early fungicidal activity.

^a^Estimated EFA values in this table are the slope of the fitted models; however, in some of the literature, EFA is often presented as −1 times the slope, so the EFA is a positive value in log_10_ colony-forming units/mL cerebrospinal fluid/day.

^b^Treatment difference = treatment − control.

#### Randomized Trial With Concurrent Cohorts Scenario

When simulating randomized trials with concurrent treatment cohorts, the 2-step and LMM approaches produce very similar estimates of treatment difference in EFA (Table 2). Both approaches exhibit a very similar performance in the calculation of treatment difference in EFA when log_10_ quantitative culture values are simulated using the multivariate normal distribution. Whether the cohort-level EFA estimates are null (Case 1) or not (Case 2), both approaches produce comparably narrow CIs with type I error rates and coverage probabilities near the nominal level. When simulated data are generated using the multivariate lognormal distribution (Case 3), the 2-step and LMM approaches produce nearly identical estimates of average treatment difference in EFA, but the narrower CI associated with the LMM results in a more efficient estimate.

Unlike the historical benchmark scenario, power simulations for the comparison of concurrent cohorts do not illustrate a consistent loss of power in the LMM (Figure 2*E*–*H*). A very similar trajectory of power is observed in both approaches across varied conditions and distributions of data. However, the LMM demonstrates greater power than the 2-step approach, regardless of sample size or the magnitude of the treatment difference, when the multivariate lognormal distribution is used to simulate data with outliers (Figure 2*G* and 2*H*). Across all concurrent control scenarios, the LMM produces an attenuated estimate of EFA compared to the 2-step approach (Supplementary Table 2).

#### Impact of Early Sterility

In all simulated cases of early sterility, the LMM produces a biased estimate of EFA (Supplementary Figure 1). The magnitude of this bias increases substantially in datasets with a high percentage of early sterility. While the 2-step approach is less impacted by missingness created through the removal of repeat sterile CSF cultures, it is still an imperfect estimator.

### Real Data Analysis

**Figure 3.**
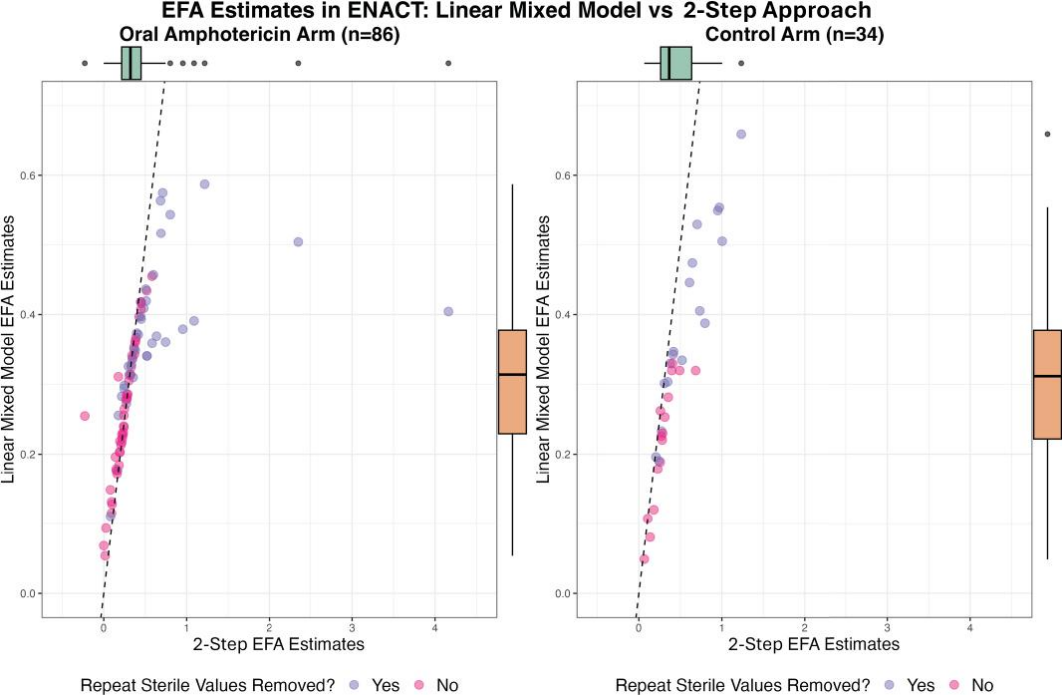
Box-scatterplot of participant-level early fungicidal activity (EFA) estimates from the ENACT study: linear mixed model (LMM) approach vs 2-step approach by linear regression. The EFA estimates from both methods were calculated with the log_10_ colony-forming units/mL/day. The left panel has EFA estimates for participants in the combined oral amphotericin arm. The right panel has EFA estimates for participants from the control arm. Across both panels, there is a nearly 1:1 relationship between the 2 estimates in participants who did not reach sterility. Participants who reached sterility tend to have a steeper 2-step estimate than the LMM estimate.

The 2-step and LMM approaches were each fitted to the ENACT trial data and used to analyze EFA on the treatment group level. Of the 141 ENACT participants, 21 had sterile lumbar punctures at baseline and were omitted from the analysis. The remaining 120 participants who were nonsterile at baseline were used in the real data analysis: 86 participants in the combined experimental oral amphotericin arm and 34 participants in the control arm (Supplementary Figure 2, Figure 3). When implementing the 2-step approach, the cohort-level EFA for nonsterile ENACT participants is 0.42 (95% CI, .31–.53) in the combined experimental arm and 0.46 (95% CI, .36–.55) in the control arm, with an estimated treatment difference of −0.04 (95% CI, −.18 to .11) log_10_ CFU/mL/day (Supplementary Table 3). The LMM estimate of treatment group EFA is 0.31 (95% CI, .27–.35) in the experimental cohort and 0.37 (95% CI, .31–.44) in the control cohort, with an estimated treatment difference of −0.06 (95% CI, −.14 to .02) log_10_ CFU/mL/day (Supplementary Table 3). While the point estimates of EFA within ENACT treatment groups produced by the LMM are attenuated compared to those produced by the 2-step approach, both approaches produce estimates of treatment difference close to the null with comparably narrow CIs.

From a box-scatterplot of participant-level EFA estimates in the ENACT oral amphotericin cohort, the distribution of EFA estimates produced by the 2-step approach is heavily right-skewed (Figure 3). On the other hand, EFA estimates produced by the LMM approach are more evenly distributed due to the LMM enforcing a normal distribution of its random slopes. We also observe a nearly 1:1 relationship between the 2-step and LMM estimates of EFA in participants who did not reach sterility. However, participants who reached sterility—that is, participants whose repeated sterile observations were removed for the analysis—are associated with overall steeper estimates of EFA with the 2-step approach than the LMM approach, and there is a departure from this 1:1 relationship between approaches.

## DISCUSSION

Our systematic review of cryptococcal literature revealed a consistent discrepancy between EFA estimates produced by the 2-step approach and the LMM, suggesting that there is meaningful difference in the 2 approaches that may impact clinical interpretation. Therefore, EFA estimates should not be interpreted or compared across studies without accounting for the underlying statistical models and data preprocessing. And we also recommend documenting statistical methods and data preprocessing steps when reporting EFA estimates in cryptococcal meningitis publications.

Through simulations of a single-arm cryptococcal trial, we showed that the LMM produces an estimate of EFA that is attenuated compared to the estimate produced by the 2-step approach and biased toward the null. The 2-step approach consistently outperformed LMM under this scenario. On the other hand, simulations of randomized trials with concurrent control cohorts revealed that calculating a treatment difference between 2 treatment arms can cancel out the bias in the cohort-level EFA estimates under certain scenarios, allowing for the LMM to produce a more efficient estimate, especially in cryptococcal data with outliers. These observations are consistent with the results of our real data analysis, where cohort-level estimates of EFA produced by the LMM were lower than those from the 2-step approach, though both approaches produced comparable estimates of treatment difference in EFA. When comparing participant-level EFA estimates from the 2 approaches, there are more discrepancies between the methods among participants who achieved early sterility. Our simulations assessing the impact of early sterility illustrate the bias in EFA estimates from both the 2-step and LMM approaches in the presence of high proportion early sterility, though the LMM suffered from increasingly larger bias as the percentage of missingness increased.

Historically, cryptococcal meningitis trial participants were not always expected to achieve sterility within the first 14 days of treatment [45]. However, recent advances in screening and combination antifungal therapy have made it increasingly common for trial participants to achieve sterility even within 7 days. As a result, fewer culture data points can be utilized for assessing EFA. This poses challenges and concerns for both methods, especially the LMM. The common LMM fits a normal distribution for the random effects, which shrinks outliers and extreme values toward the population average. The LMM also implicitly downweights individuals with fewer data points, who in fact tend to have a steeper slope. The missingness of CSF culture due to sterility is non-ignorable and violates the missing at random assumption for LMM. For these reasons, LMM produces biased estimates of both individual-level and cohort-level EFA in the presence of early sterility. This limitation of the LMM has a key clinical relevance: Whereas clinical data suggest that early sterilization is associated with better outcomes in cryptococcal meningitis trial participants, based on the statistical evidence in this study, early sterilization could yield less reliable results in LMM and may undermine findings of an effective treatment.

While not subject to the above limitations like the LMM, the 2-step approach also has limitations when handling early sterility. In the 2-step approach, each individual simple linear regression only relies on the CSF cultures from that participant. Having <3 data points can lead to highly unstable estimate of the slope, and the estimates become sensitivity to the timing of the lumbar puncture. Time to sterility, with the timing of the lumbar puncture handled via interval censoring, may be considered as an alternative endpoint.

Based on our findings across literature review, simulation studies, and real data analysis, the 2-step approach is preferred for estimating EFA in cryptococcal meningitis studies, especially when early culture sterility is common. EFA estimates should not be interpreted or compared across studies without accounting for the underlying statistical models and data preprocessing. With the advancement of antifungal treatment, how to handle and account for early sterility becomes a challenge in evaluating early fungal clearance.

## Supplementary Material

ofag159_Supplementary_Data

## Supplementary Data

Supplementary materials are available at *Open Forum Infectious Diseases* online. Consisting of data provided by the authors to benefit the reader, the posted materials are not copyedited and are the sole responsibility of the authors, so questions or comments should be addressed to the corresponding author.
