# Supplementary material for: Assessing Statistical Methods for Estimating the Cerebrospinal Fluid Early Fungicidal Activity as an Endpoint for Cryptococcal Meningitis Clinical Trials: A Systematic Review and Simulation Study
Source: Open Forum Infect Dis. 2026 Mar 22;13(4):ofag159. doi: 10.1093/ofid/ofag159 (PMC13064499; doi:10.1093/ofid/ofag159)

## **Supplemental Tables**

**Table S1: Key Findings of Systematic Early Fungicidal Activity Literature Review**

| Study | Treatment Group(s) | N | EFA via Two-Step Approach (95% CI)  log_10_ CFU/mL/day | EFA via LMM Approach (95% CI)  log_10_ CFU/mL/day |
| --- | --- | --- | --- | --- |
| Boulware et al, 2014 | AmB + FLU | 189 | 0.36 (0.32, 0.40) | 0.31 (0.28, 0.33) |
| Molloy et al, 2018 | Treatment Difference:  FLU + 5FC vs. 2-Wk AmB | 182 vs 179 | 0.16 (0.12, 0.21) | 0.10 (0.07, 0.13) |
|  | Treatment Difference:  1-Wk AmB vs. 2-Wk AmB | 179 vs 182 | 0.02 (-0.02, 0.07) | 0.01 (−0.01, 0.04) |
| Rhein et al, 2019 | AmB + FLU + sertraline | 229 | 0.43 (0.37, 0.50) | 0.33 (0.30, 0.36) |
|  | AmB + FLU + placebo | 231 | 0.47 (0.40, 0.54) | 0.33 (0.30, 0.35) |
| Jarvis et al, 2022 | AmB daily + 5FC | 381 | 0.44 (0.03, 0.81) | 0.42 (0.16, 0.67) |
|  | Liposomal AmB + FLU + 5FC | 363 | 0.41 (0.04, 0.78) | 0.40 (0.15, 0.65) |
| Boulware et al, 2023 | Oral AmB with 2 IV Doses | 37 | 0.42 (0.29, 0.55) | 0.33 (0.27, 0.39) |
|  | All-Oral AmB | 33 | 0.40 (0.17, 0.64) | 0.25 (0.19, 0.31) |
|  | IV AmB, Controls | 34 | 0.46 (0.36, 0.55) | 0.37 (0.31, 0.44) |

AmB=amphotericin B; CI=confidence interval; EFA=early fungicidal activity; FLU= fluconazole; IV=intravenous; LMM=linear mixed model; Wk=week.

**Table S2: Power Simulation Results for Concurrent Cohorts Scenario under Multivariate Lognormal Distribution**

|  | Performance Metric | | |
| --- | --- | --- | --- |
|  | Mean  log_10_ CFU/mL/day | Median  log_10_ CFU/mL/day | CI Length |
| Case 1: n=50 | | | |
| Treatment Value | -0.299 | -0.216 | -- |
| Control Value | -0.197 | -0.109 | -- |
| True Treatment Difference^a^ | -0.102 | -0.108 | -- |
| Treatment Difference^a^: Two-Step | -0.090 | -0.095 | 0.189 |
| Treatment Difference^a^: LMM | -0.069 | -0.068 | 0.111 |
| Case 2: n=100 | | | |
| Treatment Value | -0.299 | -0.212 | -- |
| Control Value | -0.194 | -0.110 | -- |
| True Treatment Difference^a^ | -0.104 | -0.102 | -- |
| Treatment Difference^a^: Two-Step | -0.085 | -0.085 | 0.132 |
| Treatment Difference^a^: LMM | -0.063 | -0.061 | 0.078 |
| Case 3: n=150 | | | |
| Treatment Value | -0.296 | -0.211 | -- |
| Control Value | -0.198 | -0.113 | -- |
| True Treatment Difference^a^ | -0.099 | -0.098 | -- |
| Treatment Difference^a^: Two-Step | -0.085 | -0.087 | 0.108 |
| Treatment Difference^a^: LMM | -0.064 | -0.065 | 0.064 |
| Case 4: n=200 | | | |
| Treatment Value | -0.301 | -0.213 | -- |
| Control Value | -0.199 | -0.112 | -- |
| True Treatment Difference^a^ | -0.102 | -0.101 | -- |
| Treatment Difference^a^: Two-Step | -0.087 | -0.088 | 0.096 |
| Treatment Difference^a^: LMM | -0.066 | -0.066 | 0.056 |
| ^a^ Treatment difference = Treatment – Control  For the two-step approach, the 95% confidence intervals are estimated directly from the individual simple linear regression slopes. For the LMM approach, marginal slopes and corresponding standard errors of the linear mixed model are obtained using lsmeans function in R and are used to calculate a 95% confidence interval.  CI=confidence interval (95%); LMM=linear mixed model. | | | |

## **Table S3: Intercept and EFA Estimates via Two-Step and LMM Approaches in ENACT Oral Amphotericin**

|  | Oral Amphotericin Arm | | IV Amphotericin Controls | | Treatment Difference ^a^ | |
| --- | --- | --- | --- | --- | --- | --- |
| Fixed Effects | Estimate | 95% CI | Estimate | 95% CI | Estimate | 95% CI |
| Two-Step Approach | | | | | | |
| Intercept, CFU/mL | 4.28 | (3.94, 4.62) | 4.23 | (3.67, 4.79) | -- | -- |
| EFA^b^ log_10_ CFU/mL/day | 0.42 | (0.31, 0.53) | 0.46 | (0.36, 0.55) | -0.04 | (-0.18, 0.11) |
| Linear Mixed Model Approach | | | | | | |
| Intercept, CFU/mL | 4.09 | (3.76, 4.42) | 4.08 | (3.55, 4.60) | -- | -- |
| EFA^c^ log_10_ CFU/mL/day | 0.31 | (0.27, 0.35) | 0.37 | (0.31, 0.44) | -0.06 | (-0.14, 0.02) |

^a^ Treatment difference = Oral Amphotericin – Control. (Negative value favors Control).

^b^ Average value of slopes from individual simple linear regressions between log_10_ quantitative CSF culture and time of lumbar puncture (days) multiplied by -1, such that rate of CSF clearance is a positive value as log_10_ *Cryptococcus* CFU per mL CSF per day.

^c^ Estimated marginal means on the linear term from days of lumbar puncture in the linear mixed model multiplied by -1, such that rate of clearance is a positive value.

For the two-step approach, the 95% confidence intervals are estimated directly from the individual simple linear regression slopes. For the Linear Mixed Model approach, marginal slopes and corresponding standard errors of the linear mixed model are obtained using lsmeans function in R and are used to calculate a 95% confidence interval.

EFA=early fungicidal activity

## **Supplemental Figures**

## **Figure S1:** **Simulation Assessing Statistical Bias of the Two-Step approach and Linear Mixed Model approach in the presence with Early Sterility. The Two-Step approach generally centers closer to zero bias, while the Linear Mixed Model shows increasingly negative bias as proportion of early sterility increases and missingness increases. The bias of the EFA estimates were calculated with the log10 CFU/mL/day unit.**

ALT Text: The figure consists of 10 boxplots, assessing the bias of two estimation approaches—Two-Step (green) and Linear Mixed Model (orange)—across five levels of missingness caused by removing sterility data: 1%, 3%, 7%, 14%, and 23%. For each missingness level, paired boxplots display the distribution of bias around zero, marked by a dashed horizontal line.


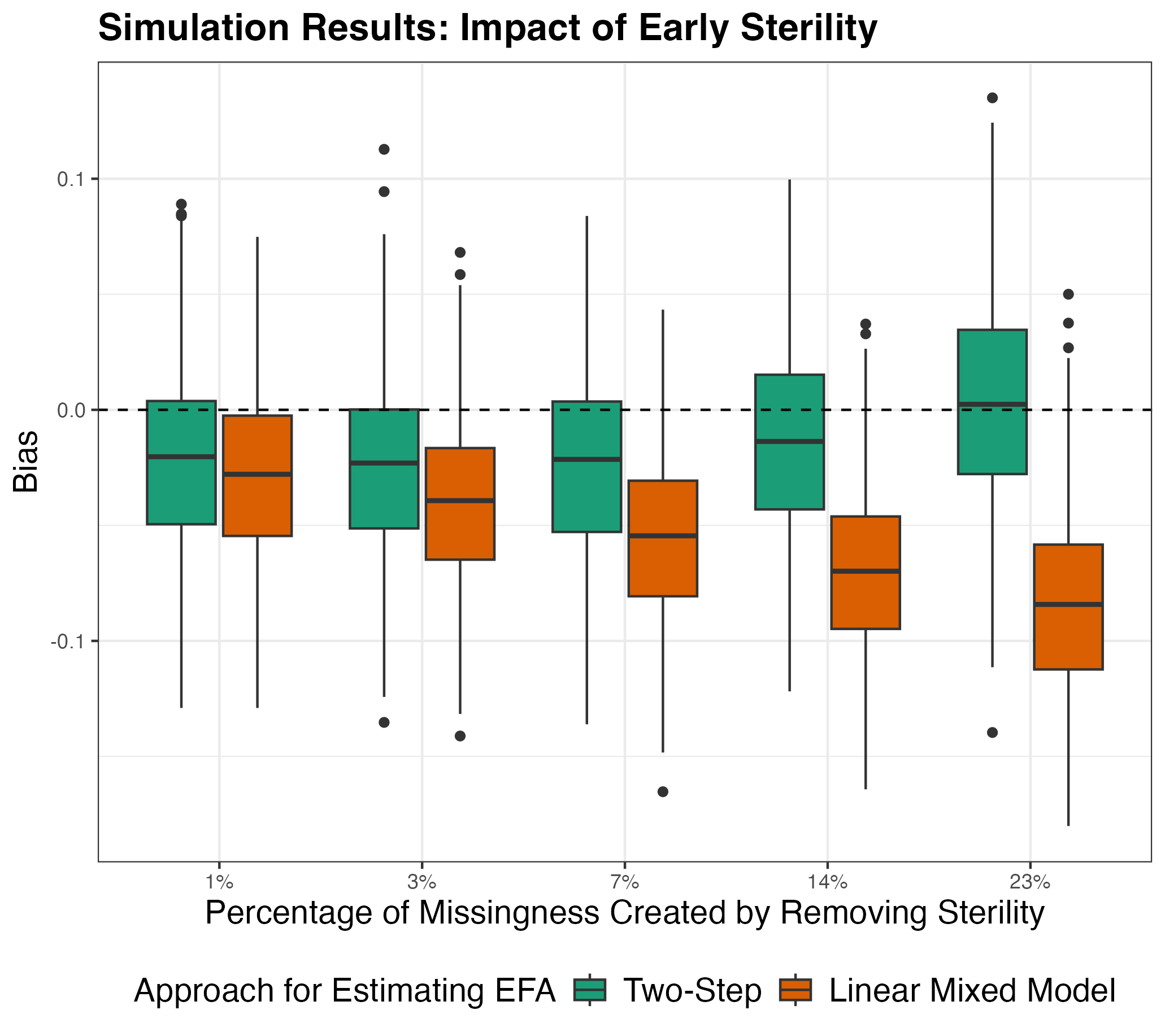


**Figure S2: Spaghetti Plot of log_10_-transformed quantitative culture over Time by Treatment Group for ENACT Participants.**

A spaghetti plot displays individual participant trajectories of log_10_ CFU/mL quantitative culture over time for ENACT trial participants. The figure is split into two panels: the left panel for the Oral amphotericin B arm with flucytosine and the right panel for the Control Arm receiving IV amphotericin B with flucytosine. Each panel shows many overlapping line segments representing repeated lumbar puncture measurements for individual patients. The x-axis shows time to lumbar puncture in days (0–20) and the y-axis shows log_10_-transformed quantitative culture log_10_ CFU/mL.


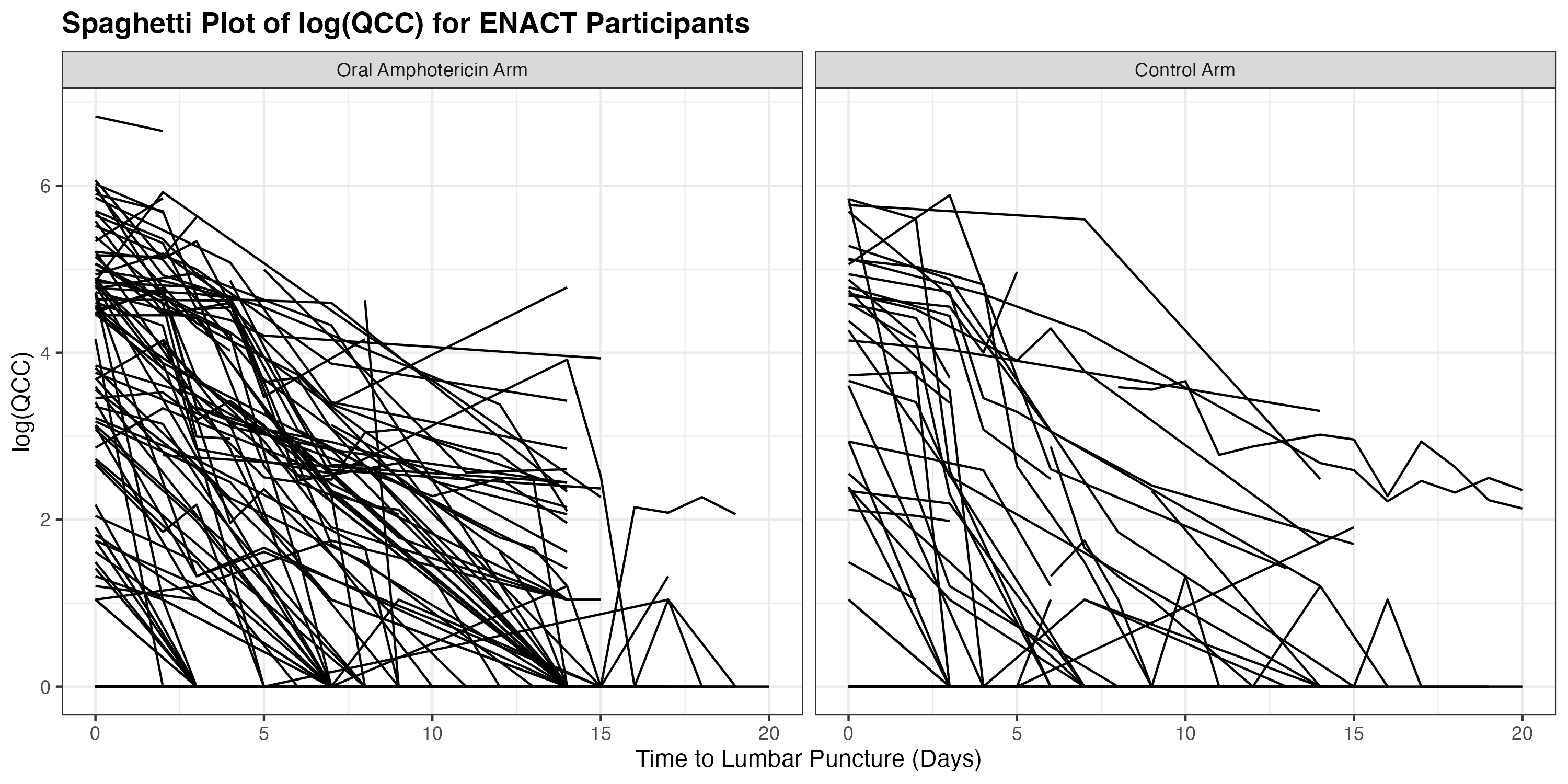

Supplement: ofag159_Supplementary_Data [file ofag159_supplementary_data.docx]
